# ITS1, 5.8S and ITS2 secondary structure modelling for intra-specific differentiation among species of the *Colletotrichum gloeosporioides sensu lato* species complex

**DOI:** 10.1186/2193-1801-3-684

**Published:** 2014-11-23

**Authors:** Sephra N Rampersad

**Affiliations:** Department of Life Sciences, Faculty of Science and Technology, The University of the West Indies, St. Augustine, West Indies, Trinidad and Tobago

**Keywords:** *Colletotrichum* spp, Internally transcribed spacer region, Secondary structure prediction

## Abstract

The *Colletotrichum gloeosporioides* species complex is among the most destructive fungal plant pathogens in the world, however, identification of member species which are of quarantine importance is impacted by a number of factors that negatively affect species identification. Structural information of the rRNA marker may be considered to be a conserved marker which can be used as supplementary information for possible species identification. The difficulty in using ITS rDNA sequences for identification lies in the low level of sequence variation at the intra-specific level and the generation of artificially-induced sequence variation due to errors in polymerization of the ITS array during DNA replication. Type and query ITS sequences were subjected to sequence analyses prior to generation of predicted consensus secondary structures, including the pattern of nucleotide polymorphisms and number of indel haplotypes, GC content, and detection of artificially-induced sequence variation. Data pertaining to structure stability, the presence of conserved motifs in secondary structures and mapping of all sequences onto the consensus *C. gloeosporioides sensu stricto* secondary structure for ITS1, 5.8S and ITS2 markers was then carried out. Motifs that are evolutionarily conserved among eukaryotes were found for all ITS1, 5.8S and ITS2 sequences. The sequences exhibited conserved features typical of functional rRNAs. Generally, polymorphisms occurred within less conserved regions and were seen as bulges, internal and terminal loops or non-canonical G–U base-pairs within regions of the double stranded helices. Importantly, there were also taxonomic motifs and base changes that were unique to specific taxa and which may be used to support intra-specific identification of members of the *C. gloeosporioides sensu lato* species complex.

**Electronic supplementary material:**

The online version of this article (doi:10.1186/2193-1801-3-684) contains supplementary material, which is available to authorized users.

## Introduction

*Colletotrichum gloeosporioides* is one of the most ubiquitous fungal plant pathogens in the world (Sutton 1992; Cannon et al., 2008) and has been associated with at least 1,972 different fungus-host combinations in the fungal databases (http://nt.ars-grin.gov/fungaldatabases/) including many tropical fruit crops (See Phoulivong et al. 2010 for a review of *Colletotrichum* species infecting tropical fruits). It has also been established that *C. gloeosporioides* is a species complex (*Colletotrichum gloeosporioides sensu lato;* Weir et al. 2012). Non-molecular traits commonly used to assign intra-specific ranking to these segregate taxa do not demonstrate adequate variability (e.g. using morphological characters), and/or are homoplasious (e.g. morphology and host range criteria). In view of this difficulty, there is a preference among many practitioners to refer to the broad, group-species concept rather than referring to names at the intra-specific level. However, correct identification is important for biosecurity and quarantine reasons, and for development of more targeted integrated disease management schemes.

To date, multi-locus phylogeny must be used for correct identification of *Colletotrichum* species (Weir et al. 2012). There are several genetic markers that are currently used for member species assignment including partial Actin (ACT), Calmodulin (CAL), Glutamine synthetase (GS), Glyceraldehyde 3-phosphate dehydrogenase (GAPDH), β-Tubulin (TUB2), Apn2/Mat and the nuclear rDNA internally transcribed spacer (ITS) region (Silva et al. 2012; Weir et al. 2012). However, some of the problems encountered with this approach include (i) some isolates still had ambiguous phylogenetic placement based on separate gene tree assessment or when a concatenated data set was used (Weir et al. 2012, (ii) other isolates are recalcitrant to amplification (e.g. CAL primers) and/or multiple bands are produced after PCR amplification which requires gel extraction and purification prior to sequencing (Weir et al. 2012), and (iii) there is information bias among the different genes used to identify member species of this complex (Rampersad et al. 2014).

The nuclear internal transcribed spacer (ITS) regions have been used as molecular markers because of their relative variability and ease of PCR amplification (Nilsson et al. 2012). The ITS array consists of the entire ITS1, 5.8S and ITS2 regions of the nuclear rDNA cistron. It is a multigene family with the potential for variation among tandem repeats. Polymorphisms are not uniformly distributed across the ITS array. The 5.8S gene sequence is highly conserved but the ITS1 and ITS2 sequences are more variable and are highly polymorphic depending on the fungal species (Hillis and Dixon 1991; Coleman 2007; Nilsson et al. 2008). Evidence suggests that significant variation among ITS sequences is found only within organisms that are diploid or polyploid hybrids, and of disparate parents (Buckler 1997). It is believed that concerted evolution allows homogenization of the many copies of this array and it is proposed that the ITS can be analyzed as a single gene (Coleman 2003). ITS sequences are typically found to be more similar within species and more divergent between species (Alvarez and Wendel 2003). In addition to being widely used for phylogenetic inference and in systematics, the ITS region is the formal fungal barcode and is the primary choice for molecular identification of fungi from a number of sources (Schoch et al. 2012). The difficulty in using ITS sequences for phylogenetic inference, however, is appropriate ITS sequence alignment which must be carried out in the absence of a translated protein product (Coleman 2009). Further, many intergenic spacers may exist as a mosaic of functional elements and inactivated pseudogenes at different stages of decay (Degnan et al. 2011). In addition, it is important that the presence of chimeric sequences be ascertained prior to sequence alignment.

Immediately post-transcription, the initial ITS transcript folds and forms helices that provide recognition and docking signals that enable processing of the transcript into mature rRNAs (van Nues et al. 1995; Joseph et al. 1999; Venema and Tolervey 1999). Schlötterer et al. (1994) found that the more variable portions of ITS2 appear to be slow evolving, at a rate close to neutral which suggests no selection. The relatively conserved regions of the ITS2 sequence are stabilized by selective forces which ensure correct rRNA processing (Coleman 2009). Less information about the function and the secondary structure of ITS1 is available, however, the region may play a role in the maturation of the 18S rRNA (van Nues et al. 1994, 1995; Coleman 2003, 2007). Although the ITS1 and ITS2 sequences can vary significantly at the sequence level, the sequences still display high levels of conservation at the structural level (Hausner and Wang 2005).

Secondary structure prediction is advantageous for species identification because it allows for the detection of sequencing errors, pseudogenes and genetic footprints indicative of past hybridization events (Coleman 2009). Accordingly, structural information can offer supplementary information for species identification (Coleman 2003, 2007). Non-functional pseudogenes are readily recognizable by their irregular 5.8S sequences and by the absence of some or all of the relatively conserved regions of ITS2, can be determined through the use of secondary structure data (Freire et al. 2012). In structure modelling, however, sequence read errors can give rise to artificial structures involving several-to-many base-pairs, whereas they only give rise to single-base-pair alignment mismatches in similarity searches.

Conservation of certain domains and nucleotide motifs are apparent across the eukaryotic kingdom (Coleman 2007). By analyzing the predicted secondary structure of an rRNA sequence and detecting conserved domains and motifs, it is possible to estimate whether the sequence is likely to code for functional rRNA and as such, validate the authenticity of rDNA gene copies (Harpke and Peterson 2008). Therefore, invalid ITS sequences that would otherwise negatively affect phylogenetic reconstruction can be removed from the data set. Additionally, given the number of ITS sequences that are misidentified and mislabelled in International Nucleotide Sequence Databases (INSD: GenBank, ENA, and DDBJ; Nilsson et al. 2012; Crouch et al. 2009) it is important that sequences be validated prior to use in subsequent analyses (Nilsson et al. 2012; Schoch et al. 2014).

This is the first study to systematically investigate the potential use of ITS1, 5.8S and ITS2 consensus secondary structure prediction towards species identification within the *C. gloeosporioides sensu lato* species complex based on type and query sequences. The main objectives of this study were: (i) to assess the nature of polymorphisms (the number and type) that may accumulate in the ITS1 and ITS2 rDNA sequences; (ii) to detect whether sequences under study are pseudogenes or represent PCR artifacts as a result of replication or sequencing errors; (iii) to examine the predicted consensus secondary structures for all epitypes and query isolates for separate ITS1, 5.8S and ITS2 markers and identify ITS structural features, including conserved motifs and variable regions; (iv) to determine whether predicted ITS secondary structures can be used to identify species within the *C. gloeosporioides sensu lato* species complex and to discuss the usefulness of secondary structure analyses to validate ITS sequence data for use in phylogenetic reconstruction.

## Results and discussion

### ITS sequence analysis and predicted secondary structures

The trimmed and edited sequences of each marker were as follows: ITS1, 140 nucleotides; 5.8S, 117 nucleotides and ITS2, 164 nucleotides. MAFFT analysis (http://mafft.cbrc.jp/alignment/server/ (Katoh 2005, 2008) revealed no evidence of chimeric sequences present in the ITS data set and indicated good quality ITS sequences with no stochastic or artifactual nucleotide data.

### ITS1 structure modelling

**Figure 1:**
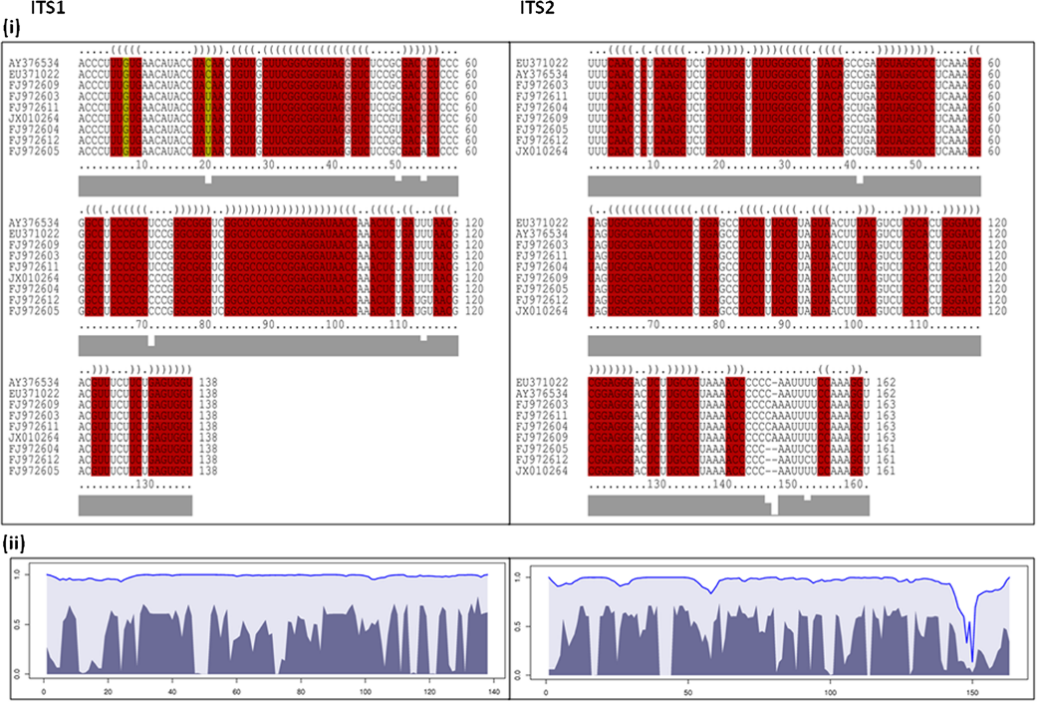
**Column alignment and STAR (structure-based alignment reliabilities) profile plots for ITS1 and ITS2 sequences as generated by LocaRNA-P.** (i): LocaRNA-P-inferred (i): Column alignment -Dot-Bracket Notation (DBN) - Dotted positions represent unpaired bases and matching parenthesized positions represent paired bases, and (ii): a STAR (structure-based alignment reliabilities) profile plots for ITS1 and ITS2 sequences. In the probability plots, the dark regions indicate structure reliability, the light regions represent sequence reliability, and the thin line shows the combined column-reliability. The STAR profile plot combined with automated detection of high-reliability regions yields accurate boundaries of structural RNA.

**Figure 2:**
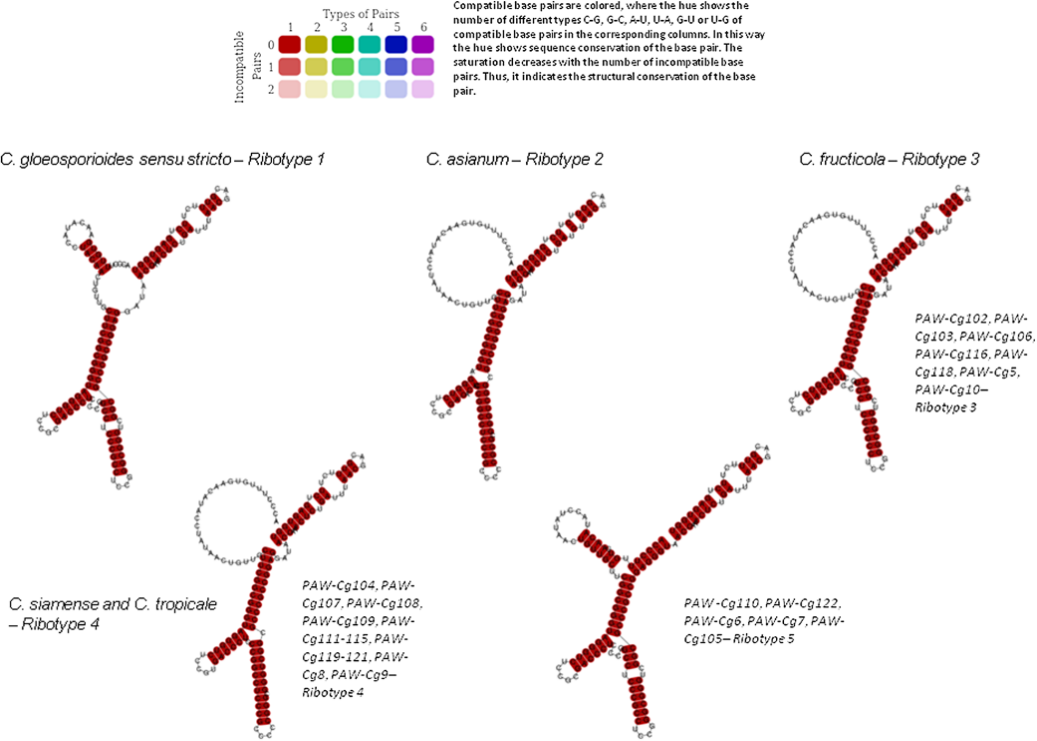
**Consensus minimum free energy (MFE) structures and proposed ribotypes for ITS1 marker according to individual species of the** ***C. gloeosporioides sensu lato*** **species complex and query sequences.**

RNA sequences of the ITS1 and ITS2 markers were aligned using the align and fold approach (Figure 1). The consensus minimum free energy (MFE) structures for the ITS1 marker according to species are illustrated in Figure 2. The delta G required for formation of the secondary structures was on average -55.1 kcal/mol. The GC content of the ITS1 sequences was on average 56.7%. Within the entire data set, there were 24 polymorphic sites, 19 singleton sites and 12 indel sites with two indel haplotypes. The indel haplotype diversity was calculated to be 0.054. Nucleotide diversity (Pi) was calculated as 0.02296 ± 0.00794. Total number of mutations (Eta) was 27. When only type and ex-type sequences were considered, there were five polymorphic sites and five mutations. A comparison of predicted structures for each sequence revealed deviations from the consensus secondary structure of the ITS1 region of *C. gloeosporioides sensu stricto* (Figure 2). Overall, four ITS1 ribotypes were proposed based on variations in type sequences: Ribotype 1 – *C. gloeosporioides sensu stricto*, Ribotype 2: *C. asianum*, Ribotype 3: *C. fructicola*, Ribotype 4: *C. siamense* and *C. tropicale* (Figure 2).

The consensus secondary structure of ITS1 marker of *C. gloeosporioides sensu stricto* consisted of a long, double helix; at its central part, the double helix contained one large, internal loop in addition to other asymmetrical internal loops. There were three non-canonical G-U base pairings and a number of base substitutions. Non-canonical G–U pairing presents certain degeneracy in base-pairing which may provide structural flexibility and can be allowed within rRNA secondary structures without resulting in significant structural changes (Mullineux and Hausner 2009). Comparisons of consensus structures suggest that insertions/deletions that impact upon helix length or base changes that occur in loops or bulges do not necessarily affect the formation of mature functional rRNA and these regions may be susceptible to such changes (Bridge et al., 2008).

There is no consensus structure for this marker across all eukaryotes, however, the GGCRY-RYGYC motif was found in the ITS1 sequence alignment and has been identified as similar to the inverted repeats found in ascomycetes and to the stem of helix 1C in some angiosperms (Liu and Schardl 1994).

**Figure 3:**
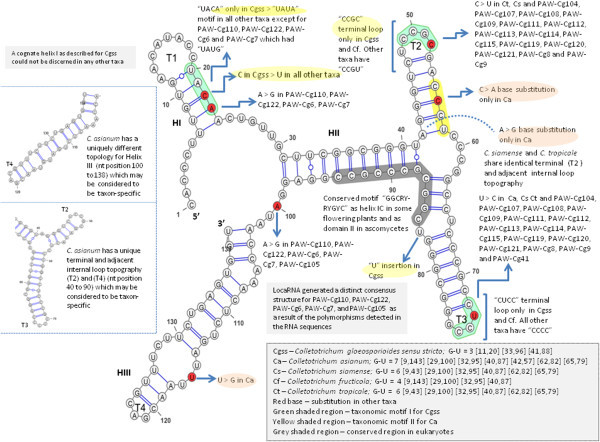
**The predicted consensus secondary structure of the ITS1 marker mapped onto** ***C. gloeosporioides sensu stricto*** **(type sequence EU371022) whose structure was generated by the LocaRNA-P pipeline.**

There was one taxonomic motif unique to *C. gloeosporioides sensu stricto* that characterized the second internal loop in the predicted structure which was identified as “UACA” (Figure 3). All other taxa had “UAUA” except for isolates PAW-Cg110, PAW-Cg122, PAW-Cg6 and PAW-Cg7 which had “UAUG”. There was also a unique taxonomic motif in the secondary structure predicted for *C. asianum* identified as “CACU” but exists as “CCCU” in all other taxa. *C. fructicola* had differences in the internal loop and terminal loops T2 and T3. *C. siamense* and *C. tropicale* shared more structural similarities than with any of other species. *C. asianum* had a unique structure that appeared to be taxon-specific.

As a result of the number of polymorphisms in sequences of PAW-Cg110, PAW-Cg122, PAW-Cg6 and PAW-Cg7, the predicted consensus secondary structure was distinct from all other taxa and a fifth ribotype was proposed. Among eukaryotes, there is an apparent variability in the number of helices and structural details that occur in the ITS1 transcript, for example, four helices were identified in *Chlorobionta* (Coleman et al. 1998; Gottschling et al. 2001) and *Saccharomyces cerevisiae* (van Nues et al., 1994), but seven helices were proposed for *Digenea* (von der Schulenburg et al., 1999). Since there is no consensus structure for this ITS marker as there is for the ITS2 marker, it is difficult to determine whether these variants are normally distributed or may be representative of a separate species within the *C. gloeosporioides sensu lato* complex but which was not considered in this study.

### 5.8S structure modelling

There were no polymorphic sites within the aligned 5.8S sequences and the entire 117 nt region was conserved with 0.0000 entropy (Hx) value. The delta G required for formation of the secondary structure of the 5.8S gene was -15.2 kcal/mol, and the GC content was 39.3%.

**Figure 4:**
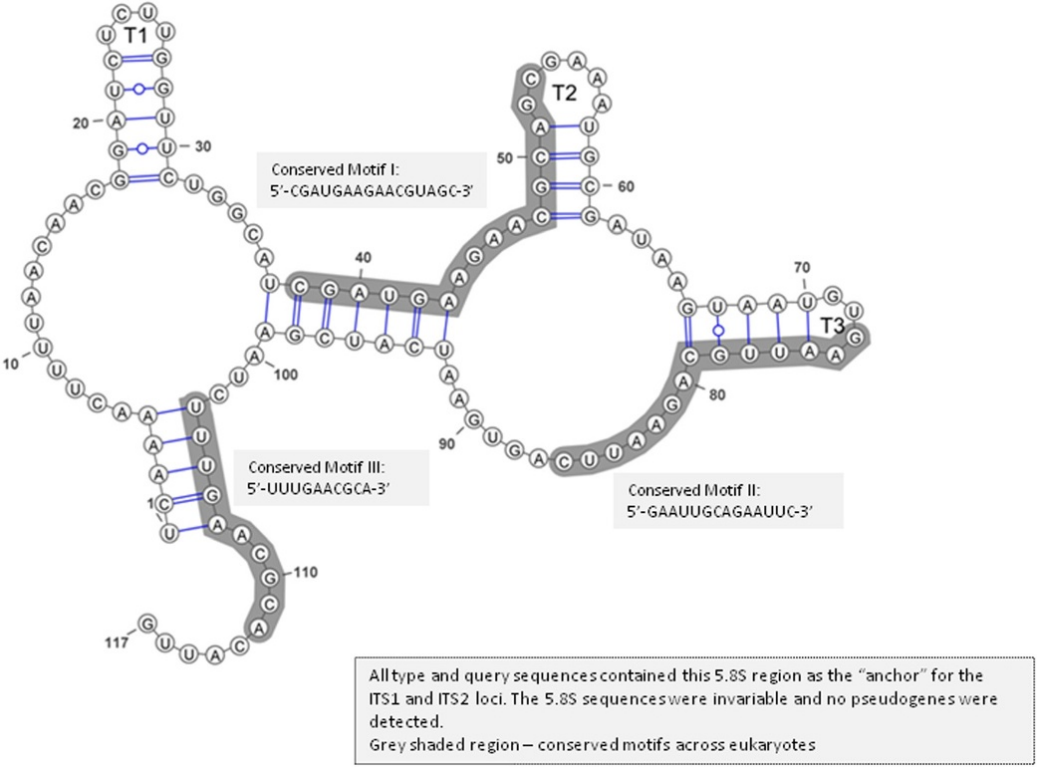
**The predicted consensus secondary structure of the 5.8S region which was invariable across all sequences analysed in the study.**

Conserved motifs for the 5.8S gene are not widely reported or described in fungi. However, at least three motifs of the 5.8S gene are conserved among angiosperms: Motif I: 5′-CGAUGAAGAACGUAGC-3′ (Harpke and Peterson 2008); Motif II: 5′-GAAUUGCAGAAUCC-3′ (Jobes and Thien, 1997); Motif III: (5′-UUUGAACGCA-3′) (Harpke and Peterson, 2008). All three motifs were identified in the 5.8S aligned sequences with the exception of a single base substitution (U > C) in Motif II: 5′-GAAUUGCAGAAUUC-3′ (Figure 4). In the 5.8S DNA sequence, Motif II contains an EcoRI restriction site (GAA/TTC) which is conserved in fungi and may serve to discriminate between cognate Motif II sequences in fungi and angiosperms (Jobes and Thien 1997). The presence of all three motifs in each sequence of the 5.8S alignment indicated that no pseudogenes were included in the data set. A search of the Rfam database (http://rfam.xfam.org/; Burge at al., 2012) resulted in a sequence match (bit score =172.3, error value =1.1e-47) for 5.8S rRNA-RF00002 Accession given in the Rfam database. The 5.8S rRNA plays a critical role in ribosome movement and in protein translation and as such, displays a high degree of pan-eukaryotic conservation (Abou-Elela and Nazar 1997).

### ITS2 structure modelling

**Figure 5:**
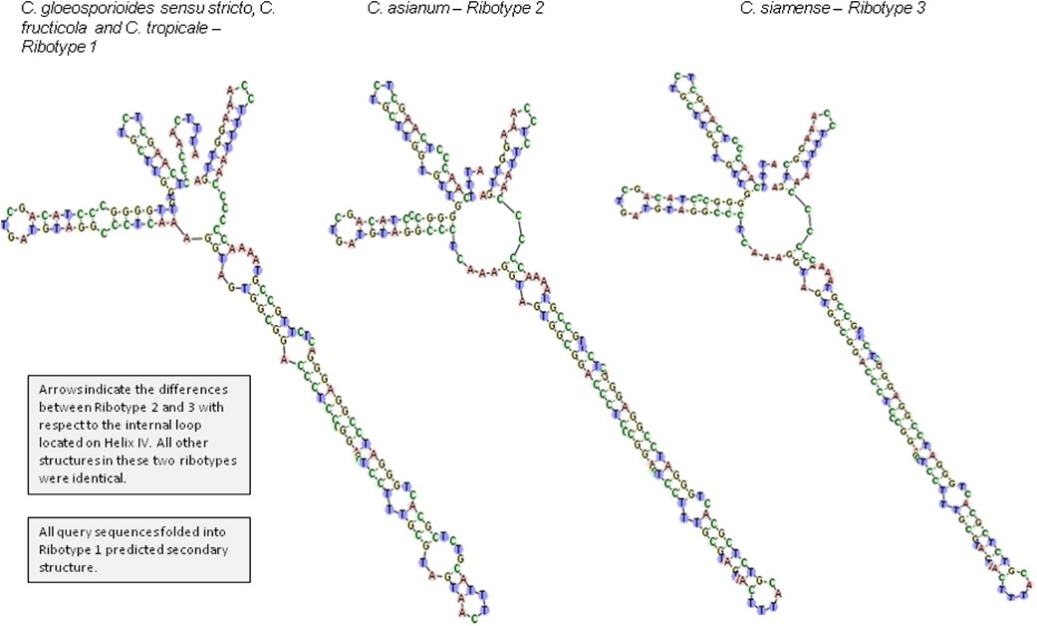
**Predicted secondary structures and proposed ribotypes for ITS2 marker according to individual species of the** ***C. gloeosporioides sensu lato*** **species complex.**

The ITS2 sequence alignment contained 27 polymorphic sites, 24 singleton sites and four indel sites in the aligned sequences of the data set with four indel haplotypes. The indel haplotype diversity was calculated to be 0.593. Nucleotide diversity (Pi) was calculated as 0.01064 ± 0.00717, which indicates a lower level of diversity to that of the ITS1 marker. The total number of mutations (Eta) was 28. When type sequences were considered, there were two polymorphic sites and two mutations. These polymorphisms ultimately gave rise to two ribotypes (Figure 5); one belonging to all taxa except for *C. asianum* and *C. siamense* species (Ribotype 1) and the other appeared exclusive to *C. asianum* and *C. siamense* species (Ribotype 2 and 3 respectively). The delta G required for formation of the secondary structures ranged from -58.59 and -52.80 (for Ribotypes 2 and 3, respectively) to -52.93 kcal/mol (for Ribotype 1). The GC content of these sequences averaged 56.17% which is very similar to that of the ITS1 marker.

Homology modelling provided high quality models and recovered a consensus secondary structure for the two ribotypes (Figure 5) which consisted of four helices radiating from a central loop. Four helices were identified and were designated I, II, III and IV. The consensus secondary structure of this region has been described as having four helices of which helix III may be the longest, and contains an “UGGU” motif 5’ to the apex. Sequence variations of this helix, such as “UGGGU”, “UGG”, or “GGU”, have been described in addition to the existence of a U-U mismatch in the second helix which is conserved in the vast majority of eukaryotes (Schultz et al. 2005; Coleman 2007). For each ribotype, percentages of helix transfer were 100% for any of the four helices. *C. asianum* and *C. siamense* had 87.14% similarity match for helix IV when ribotypes 1 and 2 (a) and (b) were compared. The ITS2 database motif description was a “U-U” mismatch (helix II, left, at 395–409 nt) “U-U” mismatch (helix II, right) with AAA between helices II and III (at 429–443 nt), “UGGU” on helix III at the 5′ end. The GC content of the ITS2 was 56.19% which was similar to that of the ITS1 marker and indicated that no pseudogenes were present in the data set.

**Figure 6:**
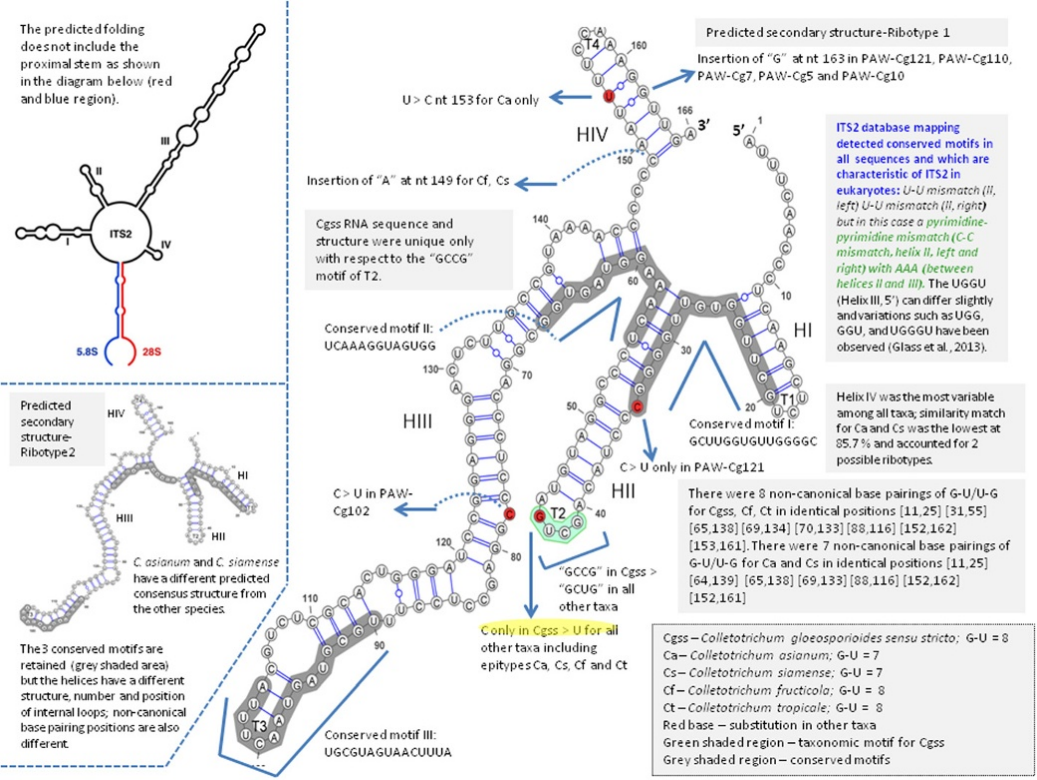
**Consensus secondary structure of the ITS2 marker mapped onto** ***C. gloeosporioides sensu stricto*** **(type sequence EU371022) whose structure was retrieved from the ITS2 database.**

The consensus structure of the ITS2 region was mapped for all type and query sequences (Figure 6). There were several unique taxonomic motifs identified in the secondary structures predicted for *C. gloeosporioides sensu stricto* especially at terminal loop 2. Similarly for *C. asianum* and *C. siamense*, there were distinct base changes, differences in internal loop number, structure and position which appeared to be taxon-specific. Slippage of RNA polymerase during transcription may result in production of mononucleotide repeats (“UUUU”) in the RNA sequences (Levinson and Gutman 1987; Hillis and Dixon 1991). These inadvertent errors in transcription may lead to an increase in the number of detected ITS2 ribotypes.

In this study, the observed variability of the ITS1 marker was higher than that of ITS2 which is in-keeping with the findings of Freire et al. (2012) and Nilsson et al. (2008). Although several conserved nucleotide sequence motifs have been identified in 5.8S and ITS2 sequences (Liu and Schardl 1994; Mai and Coleman 1997), it is the retention of functionally conserved secondary structures that enable the ITS array to play a critical role in the production of mature rRNA molecules (van Nues et al. 1994, 1995; Joseph et al. 1999; Michot et al. 1999; Venema and Tollervey 1999). It is apparent from the level of variation in the nucleotide sequence and predicted secondary structures of the ITS1 and ITS2 markers that different selective pressures may be acting at each markers. Within the ITS array, some regions are under evolutionary constraints at the level of the nucleotide sequence (Liu and Schardl 1994; Mai and Coleman 1997), while others are under positive selection at the level of the secondary structure with the emergence of concomitant compensatory base changes to preserve this structure (van Nues et al. 1994, 1995; Joseph et al. 1999).

Studies have shown that within the *C. gloeosporioides sensu lato* species complex, ITS sequences are capable of resolving approximately 50% of the accepted species (Weir et al. 2012). This reflects the low number of base changes in the ITS region across the *C. gloeosporioides sensu lato* species complex. Member species are often distinguished by only one or two base changes. In some cases, the variation in the ITS sequence is insufficient to distinguish among certain members of this species complex. There was only one other study by Bridge et al. (2008) in which the structure types of the ITS1 marker for *C. gloeosporioides* were compared. At the time of that study, many of the now-known epitype member species of this complex were not used and it is, therefore, difficult to determine the reliability of the predicted structures and precisely to which intra-specific level the identified structures belonged.

## Conclusions

This is the first study to systematically evaluate the predicted secondary structures for the rRNA sequences of member species belonging to *C. gloeosporioides sensu lato* species complex that infect papaya in Trinidad. The ITS sequences of fungal species of this complex have been considered to be insufficiently variable to reliably distinguish between member species. In this study, taxon-specific secondary structures have been predicted for certain member species of the *C. gloeosporioides sensu lato* species complex which may provide supplementary data to improve the identification of species belonging to this species complex.

## Methods

### Data sets

Type sequences of *C. gloeosporioides sensu stricto*, *C. asianum*, *C. fructicola*, *C. siamense* and *C. tropicale* (Additional file 1: Table S1) were mined from GenBank. These species within the *C. gloeosporioides* species complex have been commonly identified as the causal agents of anthracnose of tropical fruit (Phoulivong et al. 2010). In selecting the sequences from holotype and epitype specimens for analyses, there were three important considerations, (i) the type sequences were selected based on the study by Cannon et al. (2012), and were included to provide sufficient range of sequence diversity, (ii) the species used are well- characterized and recognized at the phylogenetic and morphological levels, and (iii) the selected species have been analyzed in previous independent studies.

To obtain test sequences, papaya fruit displaying symptoms of anthracnose were collected during the period 2011 to 2013 (Additional file 1: Table S2). DNA was extracted from pure single spore cultures of *Colletotrichum* sp. using the E.Z.N.A. fungal DNA extraction kit® according to the manufacturer’s instructions (Omega bio-tek Ltd., USA). The entire ITS region (496 bp) was amplified using the universal primer pair ITS4/5 (White et al. 1990) and sequenced with independent base call verification (Amplicon Express, WA, USA). Representative sequences were submitted to GenBank (KM117226 to KM117228). A total of 56 sequences were used in the final data set for generating consensus secondary structures for ITS1, 5.8S and ITS2 markers: 30 type sequences obtained from holotype and epitype specimens and 26 query sequences belonging to the *C. gloeosporioides sensu lato* complex. Other fungal sequences were also mined from GenBank (HQ238968, JF780523, EU480703, HQ238962, EF543854) and were used as out-groups to assist in defining of helical domains of the three markers based on mfold alignments in the UNAFold webserver (http://mfold.rna.albany.edu/) (Zuker 2003; Markham and Zuker 2008).

### ITS sequence analysis and alignment

Errors can occur during PCR amplification when two different DNA templates may be present. The resulting amplicon may be chimeric, that is, a mosaic of these original sequences (Jumpponen 2011). Such chimeric sequences may be misinterpreted as novel which can artificially inflate estimates of diversity and interfere with phylogenetic inference and species discrimination if undetected (Hugenholtz and Huber 2003). ITS sequences were checked for possible chimeras using the UNITE PlutoF Chimera checker (Nilsson et al. 2010; Edgar et al. 2011) and the Chimera Test developed in the Fungal Metagenomics Project at the University of Alaska (https://biotech.inbre.alaska.edu/fungal_portal/?program=chimera_test).

### Verifying ITS sequence validity

Alignments were carried out using the online version of the sequence alignment program MAFFT version 6 ((http://mafft.cbrc.jp/alignment/server/ (Katoh et al. 2005; Katoh and Toh 2008). This sequence analysis will also aid in determining if the ITS sequences were composed of stochastic, artifactual nucleotide data. The start and end point of each marker were first defined for each species using the pipeline available on the ITS2 database website (http://its2.bioapps.biozentrum.uni-wuerzburg.de/). Ultimately, three sets of sequence alignments were generated: ITS1, 5.8S and ITS2 as separate data sets.

### Comparison of GC content and nucleotide diversity of the ITS sequences

The sequence length of the ITS1 and ITS2 region for a given species can be variable, however, the two markers should have similar GC content if they are authentic sequences under functional and selective constraints and not pseudogenes (Harpke and Peterson 2008; Mullineaux and Hausner 2009). The GC content of the ITS1, 5.8S and ITS2 sequences was determined using BioEdit version 7.2.0 software. DNASP version 5.10 (Rozas et al. 2003; Librado and Rozas 2009) was used to determine the nucleotide diversity (Pi), polymorphic and singleton sites, indel sites and indel haplotypes among the ITS1, 5.8S and ITS2 sequences.

### Secondary structure prediction

Ribosomal secondary structure and motif detection was determined for the ITS2 marker. Secondary structure predictions of rRNA sequences are sensitive to single base changes which in turn, can affect hydrogen base-pairing especially along the stem aspect of a stem-loop secondary structure (Matthews et al. 2005). Consequently for this study, the ITS sequences and their electropherograms were manually reviewed and evaluated for signal quality and accurate nucleotide assignment in order to prevent user-induced errors in structural predictions. Because the core folding pattern of the ITS2 sequence is already known, this presents an external criterion or reference to check for the correctness of the predicted structures (Schultz and Wolf 2009). For the ITS2 consensus secondary structure prediction, the ITS2 database pipeline (http://its2.bioapps.biozentrum.uni-wuerzburg.de/) (Koetschan et al. 2010; Merget et al. 2012; Koetschan et al. 2012) was used. Consensus secondary structures for the ITS1 and 5.8S markers were determined using LocaRNA-P simultaneous RNA alignment and folding option of the Freiburg RNA Tools pipeline (http://rna.informatik.uni-freiburg.de:8080/LocARNA/Input.jsp) (Will et al. 2007, Will et al. 2012 and Smith et al. 2010), and the RNA folding form option of the mfold webserver using default conditions for temperature (37°C) and ionic conditions (http://mfold.rna.albany.edu/) (Zuker 2003; Markham and Zuker 2008). Consensus secondary structures for ITS1, 5.8S and ITS2 markers as radial view structures were re-drawn and annotated for publication purposes using VARNA 3.9 (Darty et al. 2009).

## Electronic supplementary material

Additional file 1: Table S1.: Authentic ITS sequences for accepted *Colletotrichum* species extracted from Canon et al. (2012). **Table S2.** Collection and isolate data. (DOCX 18 KB)

## References

[CR1] Abou -Elela S, Nazar RN (1997). Role of the 58S rRNA in ribosome translocation. Nucleic Acids Res.

[CR2] Alvarez I, Wendel JF (2003). Ribosomal ITS sequences and plant phylogenetic inference. Mol Phylogenet Evol.

[CR3] Bridge PD, Schlitt T, Cannon PF, Buddie AG, Baker M, Borman AM (2008). Domain II hairpin structure in ITS1 sequences as an aid in differentiating recently evolved animal and plant pathogenic fungi. Mycopathologica.

[CR4] Burge SW, Daub J, Eberhardt R, Tate J, Barquist L, Nawrocki EP, Eddy SR, Gardner PP, Bateman A (2012). Rfam 110: 10 years of RNA families. Nucleic Acids Res.

[CR5] Buckler ES, Ippolito A, Holtsford TP (1997). The evolution of ribosomal DNA: divergent paralogues and phylogenetic implications. Genetics.

[CR6] Cannon PF, Buddie AG, Bridge PD (2008). The typification of *Colletotrichum gloeosporioides*. Mycotaxon.

[CR7] Cannon PF, Damm U, Johnston PR, Weir BS (2012). *Colletotrichum*–current status and future directions. Stud Mycol.

[CR8] Coleman AW, Preparata RM, Mehrotra B, Mai JC (1998). Derivation of the secondary structure of the ITS-1 transcript in *Volvocales* and its taxonomical correlations. Protist.

[CR9] Coleman AW (2003). ITS2 is a double-edged tool for eukaryote evolutionary comparisons. Trends Genet.

[CR10] Coleman AW (2007). Pan-eukaryote ITS2 homologies revealed by RNA secondary Structure. Nucl Acids Res.

[CR11] Coleman AW (2009). Is there a molecular key to the level of “biological species” in eukaryotes? A DNA guide. Mol Phylogenet Evol.

[CR12] Crouch JA, Clarke BB, Hillman BI (2009). What is the value of ITS sequence data in *Colletotrichum* systematics and species diagnosis? A case study using the falcate-spored graminicolous *Colletotrichum* group. Mycologia.

[CR13] Darty K, Denise A, Ponty Y (2009). VARNA: Interactive drawing and editing of the RNA secondary structure. Bioinformatics.

[CR14] Degnan PH, Ochman H, Moran NA (2011). Sequence conservation and functional constraint on intergenic spacers in reduced genomes of the obligate symbiont *Buchnera*. PLoS Genet.

[CR15] Edgar RC, Haas BJ, Clemente JC, Quince C, Knight R (2011). UCHIME improves sensitivity and speed of chimera detection. Bioinformatics.

[CR16] Freire MCM, Roméria da Silva M, Zhang X, Almeida AMR, Stacey G, de Oliveira LO (2012). Nucleotide polymorphism in the 58S nrDNA gene and internal transcribed spacers in *Phakopsora pachyrhizi* viewed from structural models. Fungal Genet Biol.

[CR17] Gottschling M, Hilger HH, Wolf M, Diane N (2001). Secondary structure of the ITS1 transcript and its application in a reconstruction of the phylogeny of *Boraginales*. Plant Biol.

[CR18] Harpke D, Peterson A (2008). 5.8S motifs for the identification of pseudogenic ITS regions. Botany.

[CR19] Hausner G, Wang X (2005). Unusual compact rDNA gene arrangements within some members of the *Ascomycota*: evidence for molecular co-evolution between ITS1 and ITS2. Genome.

[CR20] Hillis DM, Dixon MJ (1991). Ribosomal DNA: molecular evolution and phylogenetic inference. Q Rev Biol.

[CR21] Hugenholtz P, Huber T (2003). Chimeric 16S rDNA sequences of diverse origin are accumulating in the public databases. Int J Syst Evol Microbiol.

[CR22] Jobes DV, Thien LB (1997). A conserved motif in the 58S ribosomal RNA (rRNA) gene is a useful diagnostic marker for plant internal transcribed spacer (ITS) sequence. Plant Mol Biol Rep.

[CR23] Joseph N, Krauskopf E, Vera MI, Michot B (1999). Ribosomal internal transcribed spacer 2 (ITS2) exhibits a common core of secondary structure in vertebrates and yeast. Nucl Acids Res.

[CR24] Jumpponen A (2011). Analysis of ribosomal RNA indicates seasonal fungal community dynamics in Andropogon gerardii roots. Mycorrhiza.

[CR25] Katoh K, Kuma K-I, Toh H, Miyata T (2005). MAFFT version 5: improvement in accuracy of multiple sequence alignment. Nucleic Acids Res.

[CR26] Katoh K, Toh H (2008). Recent developments in the MAFFT multiple sequence alignment program. Brief Bioinform.

[CR27] Koetschan C, Förster F, Keller A, Schleicher T, Ruderisch B, Schwarz R, Müller T, Wolf M, Schultz J (2010). T he ITS2 Database III--sequences and structures for phylogeny. Nucleic Acids Res.

[CR28] Koetschan C, Hackl T, Müller T, Wolf M, Förster F, Schultz J (2012). ITS2 database IV: interactive taxon sampling for internal transcribed spacer 2 based phylogenies. Mol Phylogenet Evol.

[CR29] Levinson G, Gutman GA (1987). Slipped-strand mispairing: a major mechanism for DNA sequence evolution. Mol Biol Evol.

[CR30] Librado P, Rozas J (2009). DNASP v5: a software for comprehensive analysis of DNA polymorphism data. Bioinformatics.

[CR31] Liu JS, Schardl CL (1994). A conserved sequence in internal transcribed spacer 1 of plant nuclear rRNA genes. Plant Mol Biol.

[CR32] Mai JC, Coleman AW (1997). The internal transcribed spacer 2 exhibits a common secondary structure in green algae and flowering plants. J Mol Evol.

[CR33] Markham NR, Zuker M, Keith J (2008). UNAFold: Software for nucleic acid folding and hybridization. Data, Sequence Analysis, and Evolution, Bioinformatics: Volume 2, Chapter 1.

[CR34] Mathews DH, Schroeder SJ, Turner DH, Zuker M, Gesteland RF, Cech TR, Atkins JF (2005). Predicting RNA secondary structure. The RNA world.

[CR35] Merget B, Koetschan C, Hackl T, Förster F, Dandekar T, Müller T, Schultz J, Wolf M (2012). The ITS2 Database. J Vis Exp.

[CR36] Michot B, Joseph N, Mazan S, Bachellerie JP (1999). Evolutionarily conserved structural features in the ITS2 of mammalian pre-rRNAs and potential interactions with the snoRNA U8 detected by comparative analysis of new mouse sequences. Nucleic Acids Res.

[CR37] Mullineux T, Hausner G (2009). Evolution of rDNA ITS1 and ITS2 sequences and RNA secondary structures within members of the fungal genera *Grosmannia* and *Leptographium*. Fungal Genet Biol.

[CR38] Nilsson RH, Kritiansson E, Ryberg M, Hallenberg N, Larsson KH (2008). Intra-specific ITS variability in the kingdom Fungi as expressed in the international sequences database and its implications for molecular species identification. Evol Bioinform.

[CR39] Nilsson RH, Abarenkov K, Veldre V, Nylinder S, De Wit P, Brosché S, Alfredsson JF, Ryberg M, Kristiansson E (2010). An open source chimera checker for the fungal ITS region. Mol Ecol Resour.

[CR40] Nilsson RH, Tedersoo L, Abarenkov K, Ryberg M, Kristiansson E, Hartmann M, Schoch CL, Nylander JAA, Bergsten J, Porter TM, Vaishampayan AJP, Ovaskainen O, Hallenberg N, Bengtsson-Palme J, Eriksson KM, Larsson HK, Larsson E, Koljalg U (2012). Five simple guidelines for establishing basic authenticity and reliability of newly generated fungal ITS sequences. Myco Keys.

[CR41] Phoulivong SA, Cai L, Chen H, McKenzie EHC, Abdelsalam K, Chukeatirote E, Hyde KD (2010). *Colletotrichum gloeosporioides* is not a common pathogen on tropical fruits. Fungal Divers.

[CR42] Rampersad SN, Hosein FN, Carrington CVF (2014). Sequence exploration reveals information bias among molecular markers used in phylogenetic reconstruction for *Colletotrichum* species. SpringerPlus.

[CR43] Rozas J, Sanchez-DelBarrio JC, Messeguer X, Rozas R (2003). DNASP, DNA polymorphism analyses by the coalescent and other methods. Bioinformatics.

[CR44] Schlötterer C, Hauser MT, von Haeseler A, Tautz D (1994). Comparative evolutionary analysis of rDNA ITS regions in *Drosophila*. Mol Biol Evol.

[CR45] Schoch CL, Seifert KA, Huhndorf S, Robert V, Spouge JL, Levesque CA, Chen W (2012). Fungal Barcoding Consortium; Fungal Barcoding Consortium Author List, Nuclear ribosomal internal transcribed spacer (ITS) region as a universal DNA barcode marker for fungi. Proc Nat Acad Sci.

[CR46] Schoch CL, Robbertse B, Robert V, Vu D, Cardinali G, Irinyi L, Meyer W, Nilsson RH, Hughes K, Miller AN, Kirk PM, Abarenkov K, Aime MC, Ariyawansa HA, Bidartondo M, Boekhout T, Buyck B, Cai Q, Chen J, Crespo A, Crous PW, Damm U, De Beer ZW, Dentinger BT, Divakar PK, Dueñas M, Feau N, Fliegerova K, García MA, Ge ZW (2014). Finding needles in haystacks: linking scientific names, reference specimens and molecular data for Fungi. Database (Oxford).

[CR47] Schultz J, Wolf M (2009). ITS2 sequence-structure analysis in phylogenetics: a how-to manual for molecular systematics. Mol Phylogenet Evol.

[CR48] Schultz J, Maisel S, Gerlach D, Muller T, Wolf M (2005). A common core of secondary structure on the internal transcribed spacer 2 (ITS2) throughout the *Eukaryota*. RNA.

[CR49] Silva DN, Talhinas P, Varzea V, Cai L, Paulo OS, Batista D (2012). Application of the Apn2/MAT locus to improve the systematics of the *Colletotrichum gloeosporioides* complex: an example from coffee (*Coffea* spp.). Mycologia.

[CR50] Smith C, Heyne S, Richter AS, Will S, Backofen R (2010). Freiburg RNA Tools: a web server integrating IntaRNA, ExpaRNA and LocARNA. Nucleic Acids Res.

[CR51] Sutton BC (1992). The genus *Glomerella* and its anamorph *Colletotrichum*. Colletotrichum.

[CR52] van Nues RW, Rientjes JMJ, van der Sande CAFM, Zerp SF, Sluiter C, Venema J, Planta RJ, Raué HA (1994). Separate structural elements within internal transcribed spacer 1 of *Saccharomyces cerevisiae* precursor ribosomal RNA direct the formation of 17S and 26S rRNA. Nucleic Acids Res.

[CR53] van Nues RW, Rientjes JMJ, Morré SA, Mollee E, Planta RJ, Venema J, Raué HA (1995). Evolutionarily conserved structural elements are critical for processing of internal transcribed spacer 2 from *Saccharomyces cerevisiae* precursor ribosomal RNA. J Mol Biol.

[CR54] Venema J, Tollervey D (1999). Ribosome synthesis in *Saccharomyces cerevisiae*. Annu Rev Genet.

[CR55] von der Schulenburg JHG, Englisch U, WaÈgele J-W (1999). Evolution of ITS1 rDNA in the *Digenea* (*Platyhelminthes*: *Trematoda*): 3′ end sequence conservation and its phylogenetic utility. J Mol Evol.

[CR56] Weir BS, Johnston PR, Damm U (2012). The *Colletotrichum gloeosporioides* species complex. Stud Mycol.

[CR57] White TJ, Bruns T, Lee S, Taylor J, Innis MA, Gelfand DH, Sninsky JJ, White TJ (1990). Amplification and direct sequencing of fungal ribosomal RNA genes for phylogenetics. PCR Protocols: A Guide to Methods and Applications.

[CR58] Will S, Reiche K, Hofacker IL, Stadler PF, Backofen R (2007). Inferring non-coding RNA families and classes by means of genome-scale structure-based clustering. PLoS Comp Biol.

[CR59] Will S, Joshi T, Hofacker IL, Stadler PF, Backofen R (2012). LocARNA-P: Accurate boundary prediction and improved detection of structural RNAs. RNA.

[CR60] Zuker M (2003). Mfold web server for nucleic acid folding and hybridization prediction. Nucleic Acids Res.

