# Supplementary material for: ITS1, 5.8S and ITS2 secondary structure modelling for intra-specific differentiation among species of the Colletotrichum gloeosporioides sensu lato species complex
Source: Springerplus. 2014 Nov 23;3:684. doi: 10.1186/2193-1801-3-684 (PMC4254888; doi:10.1186/2193-1801-3-684)
Supplement: Supplementary file 1 — Additional file 1: Table S1.: Authentic ITS sequences for accepted Colletotrichum species extracted from Canon et al. (2012). Table S2. Collection and isolate data. (DOCX 18 KB) [file 40064_2014_1384_MOESM1_ESM.docx]

**Supplementary Table S1 -** Authentic ITS sequences for accepted *Colletotrichum* species extracted from Canon et al., 2012

| **GenBank Accession** | **^1^Strain** | **Source** | **Species** |
| --- | --- | --- | --- |
| [FJ972612](http://www.ncbi.nlm.nih.gov/nuccore/FJ972612.1) | MFU 090233, ICMP 18580, CBS 130418 | Culture from holotype | *Colletotrichum asianum* |
| [FJ972605](http://www.ncbi.nlm.nih.gov/nuccore/FJ972605.1) | MFU 090233, ICMP 18580, CBS 130418 | Culture from holotype | *Colletotrichum asianum* |
| [FJ972611](http://www.ncbi.nlm.nih.gov/nuccore/FJ972611.1) | MFU090228, ICMP 18581, CBS 130416 | Culture from holotype | *Colletotrichum fructicola* |
| [FJ972603](http://www.ncbi.nlm.nih.gov/nuccore/FJ972603.1) | MFU090228, ICMP 18581, CBS 130416 | Culture from holotype | *Colletotrichum fructicola* |
| [FJ972604](http://www.ncbi.nlm.nih.gov/nuccore/FJ972604.1) | MFU 090230, ICMP 18578,CBS 130417 | Culture from holotype | *Colletotrichum siamense* |
| [FJ972613](http://www.ncbi.nlm.nih.gov/nuccore/282801678?report=fasta) | BPDI2 | Culture from holotype | *Colletotrichum siamense* |
| [JX010264](http://www.ncbi.nlm.nih.gov/nuccore/JX010264.1) | CBS 124949, ICMP18653 | Culture from holotype | *Colletotrichum tropicale* |
| AY376534 | STE-U 5297 | Culture from ex-epitype | *Colletotrichum gloeosporioides sensu stricto* |
| EU371022 | IMI 356878 | Culture from epitype | *Colletotrichum gloeosporioides sensu stricto* |
| [FJ972609](http://www.ncbi.nlm.nih.gov/nuccore/FJ972609.1) | CBS 95397 | Culture from epitype | *Colletotrichum gloeosporioides sensu stricto* |
| [GU227800](http://www.ncbi.nlm.nih.gov/nuccore/gu227800) | CBS:151 | Culture from holotype | *Colletotrichum lindemuthianum* |

**^1^Strain:**

ATCC: American type culture collection, 10801 University Boulevard, Manassas, Virginia, USA.

CBS: Culture collection of the Centraalbureau voor Schimmelcultures, Fungal Biodiversity Centre, Utrecht, The Netherlands.

ICMP: International Collection of Microorganisms from Plants, Landcare Research, Auckland, New Zealand.

IMI: Culture collection of CABI Europe UK Centre, Egham, UK.

MFU: fungarium of Mae Fah Luang University, Thailand (cultures in BCC (BIOTEC Culture Collection, Thailand).

STE-U: Culture collection of the Department of Plant Pathology, University of Stellenbosch, South Africa

**Supplementary Table S2 –** Collection and isolate data

| **Record** | **Isolate** | **County/State** | **Location** | **Cultivar** | **Year** |
| --- | --- | --- | --- | --- | --- |
| 1 | PAW-Cg5 | St. George West | North Trinidad | Red Lady | 2012 |
| 2 | PAW-Cg6 | St. George West | North Trinidad | Red Lady | 2012 |
| 3 | PAW-Cg7 | St. George West | North Trinidad | Red Lady | 2012 |
| 4 | PAW-Cg8 | St. George East | North Trinidad | Red Lady | 2012 |
| 5 | PAW-Cg9 | St. George East | North Trinidad | Tainung No. 2-F1 hybrid | 2012 |
| 6 | PAW-Cg10 | St. George East | North Trinidad | Tainung No. 2-F1 hybrid | 2012 |
| 7 | PAW-Cg102 | Victoria | South Trinidad | Tainung No. 2-F1 hybrid | 2013 |
| 8 | PAW-Cg103 | Victoria | South Trinidad | Tainung No. 2-F1 hybrid | 2013 |
| 9 | PAW-Cg104 | Victoria | South Trinidad | Tainung No. 2-F1 hybrid | 2011 |
| 10 | PAW-Cg105 | Victoria | South Trinidad | Tainung No. 2-F1 hybrid | 2011 |
| 11 | PAW-Cg106 | Victoria | South Trinidad | Tainung No. 2-F1 hybrid | 2011 |
| 12 | PAW-Cg107 | Victoria | South Trinidad | Tainung No. 2-F1 hybrid | 2011 |
| 13 | PAW-Cg108 | Victoria | South Trinidad | Red Lady | 2011 |
| 14 | PAW-Cg109 | Victoria | South Trinidad | Red Lady | 2011 |
| 15 | PAW-Cg111 | Victoria | South Trinidad | Tainung No. 2-F1 hybrid | 2013 |
| 16 | PAW-Cg112 | Victoria | South Trinidad | Tainung No. 2-F1 hybrid | 2013 |
| 17 | PAW-Cg113 | Victoria | South Trinidad | Tainung No. 2-F1 hybrid | 2013 |
| 18 | PAW-Cg114 | Victoria | South Trinidad | Tainung No. 2-F1 hybrid | 2013 |
| 19 | PAW-Cg115 | Victoria | South Trinidad | Tainung No. 2-F1 hybrid | 2013 |
| 20 | PAW-Cg116 | Victoria | South Trinidad | Tainung No. 2-F1 hybrid | 2013 |
| 21 | PAW-Cg117 | Victoria | South Trinidad | Red Lady | 2013 |
| 22 | PAW-Cg118 | Mayaro | South Trinidad | Red Lady | 2013 |
| 23 | PAW-Cg119 | Mayaro | South Trinidad | Tainung No. 2-F1 hybrid | 2013 |
| 24 | PAW-Cg120 | Mayaro | South Trinidad | Tainung No. 2-F1 hybrid | 2011 |
| 25 | PAW-Cg121 | Mayaro | South Trinidad | Tainung No. 2-F1 hybrid | 2011 |
| 26 | PAW-Cg122 | Mayaro | South Trinidad | Red Lady | 2013 |
